# Framework for automated sorting of neural spikes from Neuralynx-acquired tetrode recordings in freely-moving mice

**DOI:** 10.1186/s42234-021-00079-3

**Published:** 2021-11-23

**Authors:** Joshua J. Strohl, Joseph T. Gallagher, Pedro N. Gómez, Joshua M. Glynn, Patricio T. Huerta

**Affiliations:** 1 Laboratory of Immune & Neural Networks, Institute of Molecular Medicine, Feinstein Institutes for Medical Research, Northwell Health, Manhasset, New York, USA, 350 Community Drive, Manhasset, NY 11030 USA; 2 Department of Molecular Medicine, Zucker School of Medicine at Hofstra/Northwell, 500 Hofstra Blvd, Hempstead, NY 11549 USA

**Keywords:** Automated spike sorting, Spike clustering, MountainSort, Neuralynx, Cheetah, MATLAB, Tetrode, Electrophysiology, Mouse

## Abstract

**Background:**

Extracellular recording represents a crucial electrophysiological technique in neuroscience for studying the activity of single neurons and neuronal populations. The electrodes capture voltage traces that, with the help of analytical tools, reveal action potentials (‘spikes’) as well as local field potentials. The process of spike sorting is used for the extraction of action potentials generated by individual neurons. Until recently, spike sorting was performed with manual techniques, which are laborious and unreliable due to inherent operator bias. As neuroscientists add multiple electrodes to their probes, the high-density devices can record hundreds to thousands of neurons simultaneously, making the manual spike sorting process increasingly difficult. The advent of automated spike sorting software has offered a compelling solution to this issue and, in this study, we present a simple-to-execute framework for running an automated spike sorter.

**Methods:**

Tetrode recordings of freely-moving mice are obtained from the CA1 region of the hippocampus as they navigate a linear track. Tetrode recordings are also acquired from the prelimbic cortex, a region of the medial prefrontal cortex, while the mice are tested in a T maze. All animals are implanted with custom-designed, 3D-printed microdrives that carry 16 electrodes, which are bundled in a 4-tetrode geometry.

**Results:**

We provide an overview of a framework for analyzing single-unit data in which we have concatenated the acquisition system (Cheetah, Neuralynx) with analytical software (MATLAB) and an automated spike sorting pipeline (MountainSort). We give precise instructions on how to implement the different steps of the framework, as well as explanations of our design logic. We validate this framework by comparing manually-sorted spikes against automatically-sorted spikes, using neural recordings of the hippocampus and prelimbic cortex in freely-moving mice.

**Conclusions:**

We have efficiently integrated the MountainSort spike sorter with Neuralynx-acquired neural recordings. Our framework is easy to implement and provides a high-throughput solution. We predict that within the broad field of bioelectronic medicine, those teams that incorporate high-density neural recording devices to their armamentarium might find our framework quite valuable as they expand their analytical footprint.

## Background

Functional understanding of neural ensembles requires the ability to reliably measure the activity of single neurons, as well as to discriminate the activity of many neighboring neurons, across extended intervals of time (Buzsáki, 2004; Csicsvari, et al., 2003). In vivo electrophysiological techniques with extracellular electrodes that measure action potentials (‘spikes’) and subthreshold oscillations have been used to record activity from the brain of several mammalian species and have generated a tremendous amount of information (Cacucci et al., 2008; O'Keefe and Dostrovsky, 1971; O’Keefe and Nadel, 1976; Kunz, et al., 2021). Moreover, continuous technological advances have ensured the lasting relevance of this technique (Steinmetz, et al., 2021; van Daal, et al., 2021). Indeed, brain recordings with tetrode arrays have been adopted by many neuroscience laboratories all over the world (Yamamoto and Wilson, 2008). After obtaining a tetrode recording, spike sorting is a mandatory step for the isolation of neuronal units. This process begins by applying a band-pass filter followed by a voltage threshold to detect all events which fall into the frequency and voltage ranges containing neural spikes. Following event detection, spikes are brought into a spike sorting software package (Rey et al., 2015). Most spike sorting algorithms use dimensionality reduction techniques, such as principal component analysis (PCA), where the high dimensional features of spikes are represented in a 2-dimensional or 3-dimensional space for manual cluster cutting (Gray, et al., 1995; Quirk & Wilson, 1999). Other software packages implement a template matching approach (Laboy-Juárez, et al., 2019), and some, such as Spike2 (CED, Cambridge, UK), offer both PCA and template matching options. Events that cluster together are assigned to a unit with an arbitrary label, representing a putative neuron. During this process, clusters or events which are either poorly isolated or likely to correspond to background noise are removed from further analysis.

Since the introduction of XCLUST, a pioneering spike clustering program, by Matthew Wilson (Quirk & Wilson, 1999), several spike sorting packages have been developed using manual or semi-automated approaches. Some commonly used packages include KlustaKwik (Harris, et al., 2000), MClust (Redish et al., 2000), Offline Sorter (Plexon, Dallas, TX), and Spike2 (CED). While the manual approach has been the standard for decades, there are notable drawbacks. A significant concern is the lack of reliability, given that different manual operators can have variable outcomes and error rates can be as high as 30% (Harris, et al., 2000). The operator-dependent nature of the manual approach may thus negatively impact the objectivity and reproducibility of the sorted data (Wood et al., 2004). Another concern with manually-sorted spikes is the amount of time necessary to analyze datasets of any size. Cluster cutting is a time-intensive process, which effectively acts as a bottleneck in the analysis of acquired neural datasets. Early semi-automated algorithms have continued to rely on human intervention (Harris, et al., 2000; Hill et al., 2012), and some have shown poor accuracy (Pedreira, et al., 2012), resulting in a lasting dependence on manual techniques by the majority of the systems neuroscience community.

Recently, automated algorithms have been developed that are both sufficiently accurate and have the ability to sort data obtained from large arrays (Buccino et al., 2020; Chaure et al., 2018; Chung et al., 2017; Pachitariu et al., 2016; Rossant et al., 2016). Remarkably, these automated approaches have arisen during a period when advancements in electrode technology have enabled simultaneous recording from hundreds to thousands of densely packed recording sites, from which sorting data would be excruciatingly laborious with previously standard methods (Berényi et al., 2014; Chung, et al., 2019; Steinmetz, et al., 2021). Smaller arrays also benefit from automated algorithms, as the same approach can be applied to tetrodes for fast and objective results. MountainSort is a particularly attractive package as it has shown to be the most accurate method thus far to sort relatively low-channel-count datasets (Buccino et al., 2020; Chung, et al., 2017; Magland, et al., 2020) and, critically, it requires no user input or changing of parameters across recordings (Chung, et al., 2017).

While MountainSort has the potential to be an effective and highly useful spike sorting package, it is still in the development phase and does not have a fully integrated support platform for importing neural recordings obtained across different recording systems and setups. One software package in development to address this issue is SpikeInterface. This software is designed to import data across a variety of acquisition systems, and then send the data to any of the SpikeInterface-supported spike sorters (Buccino et al., 2020). While SpikeInterface is designed to be a unified framework for spike sorting, with a high degree of flexibility, our framework offers advantages in its simplicity. Using the framework presented in this study, the end-user can automatically merge files of recordings from the same mouse on the same day, and send many recordings into MountainSort in a single run. After sorting, spikes can be easily integrated into existing data analysis pipelines. This is all done automatically with no coding needed by the end-user. Thus, the purpose of this study is to provide a simple-to-execute framework for using MountainSort with Neuralynx-acquired neuronal data. Moreover, we validate this framework with a qualitative comparison of manually-sorted spikes against automatically-sorted spikes in neural recordings of the *Cornus Ammonis* 1 (CA1) region of the hippocampus and the prelimbic (PL) cortex in freely-moving mice.

## Methods

### Ethical statement

Animal experiments were performed in accordance with the National Institutes of Health (NIH) Guidelines under protocols approved by the Feinstein Institutes for Medical Research Institutional Animal Care and Use Committee (IACUC). Our Animal Research Program is registered with the Department of Health and Human Services (DHHS), Office of Laboratory Animal Welfare (OLAW), U.S. Department of Agriculture (USDA #21R0107), Public Health Service (PHS #A3168–01) and New York State Department of Health (NYSDOH #A-060).

### Experimental animals

All animals used in this study were male C57BL/6 mice (The Jackson Laboratory, Bar Harbor, ME) of 3 months of age. Mice were maintained on a reverse light cycle (dark: 9:00–21:00) with ad libitum access to food and water. All experiments were carried out during the dark phase of the light cycle. Prior to implanting, mice were housed in groups of four, and were single-housed after implanting. All mice were gently handled prior to surgery (15-min sessions during 3 consecutive days).

### Microdrive preparation

Custom-designed microdrive bodies were fabricated using a 3D printer (Form-2, Formlabs, Somerville, MA). The design of the microdrive body was specific to the brain region being recorded. Polyimide tubing and an electrode-interface-board (Omnetics EIB-16, Neuralynx, Bozeman, MT) were attached to each microdrive body. Tetrodes were wound from 90% platinum, 10% iridium wire (diameter 17.8 μm; California Fine Wire, Grover Beach, CA) and threaded through the polyimide tubes to create a movable 4-tetrode array (Chang, et al., 2013). On the day of implantation surgery, a ‘final cut’ of the tetrodes was made followed by electroplating with platinum black solution (Neuralynx) to an impedance under 300 kΩ.

### Surgery

All surgical procedures were performed under isoflurane anesthesia. The animal’s fur was removed from the surgical site, which was then scrubbed with betadine and isopropyl alcohol. An incision was made, exposing the skull, and a layer of C&B-Metabond (Parkell, Edgewood, NY) was applied and allowed to dry. Two craniotomies were made, one over the cerebellum and the other over the region targeted for tetrode implantation. The coordinates used for dorsal CA1 were [AP, − 2.18, ML, − 1.5] from bregma, and the coordinates for the PL cortex were [AP, + 1.98, ML, − 0.25] from bregma. After installing the ground screw into the craniotomy located in the occipital bone, the microdrive was aligned so that the tetrodes were directly above the intended region, and the microdrive was secured in place with dental acrylic. As the dental acrylic hardened, mice were given injection of buprenorphine (0.05 mg per kg) and saline (0.5 mL) subcutaneously. Implanted mice were observed for three days following surgery and provided with hydrogel cups containing meloxicam for pain relief. Tetrodes were lowered to their target depth, in CA1 or PL cortex, over the course of the next three days.

### Behavioral tasks

Neural recording during behavioral tasks were typically performed 7–10 days after the surgery. Mice with tetrodes targeted to CA1 (*n* = 4) were studied in a linear track (80 cm long). For this task, mice experienced a first ‘Run’ session (moving from one end of the track to the other 16 times; 8 runs ‘to the left’, 8 runs ‘to the right’), a ‘Rest’ period (10 min in the homecage), and a second ‘Run’ session (16 times, 8 runs ‘to the left’, 8 runs ‘to the right’), for a total of 32 runs across the length of the track. Mice with tetrodes targeted to the PL cortex (*n* = 4) were pre-trained in a T maze, before being implanted. The task consisted of running from the start point of the stem toward the decision point and then turning right, or left, to find a sweet food reward that was located at the end of the right arm. Mice were mildly food-deprived (food was removed for 3–4 h before testing), and were tested in the T maze until they reached a performance accuracy of 75%, which took 3–4 days (8 trials per day). One week after implantation, animals were tested in the T maze (8 trials in one day).

### Data acquisition

Mice were recorded using a headstage pre-amplifier (Neuralynx), which was connected to a programmable amplifier (Lynx-8, Neuralynx) and a Windows PC running the Cheetah acquisition software (Neuralynx). In this study, we used the Cheetah system and a 16-channel setup comprising of 4 tetrodes, each featuring 4 closely spaced recording channels, along with an overhead camera. The acquisition setup generated 3 datastreams: continuously-sampled neural signals, discretely-sampled spikes, and the animal’s XY location. For the continuous data, the 16 channels were acquired at 30 kHz and contained the voltage for each channel at every timestamp. For the spike data, the 16 channels were first band-pass filtered (600 Hz to 6 kHz) and only the fluctuations that surpassed the assigned voltage threshold (120 μV) were captured. For the XY data, video tracking was achieved via a ceiling-mounted camera which tracked the position of an LED (light-emitting diode) mounted to the headstage in the Cheetah software at 30 Hz. The raw video footage was separately saved as well.

### Hardware and software used for data analysis

After acquisition, data were transferred to a Linux machine (running Ubuntu 18.04.5 LTS) for sorting using the automated framework. On the Linux system, data were prepared for sorting with MountainSort, using MATLAB 2017b (MathWorks, Natick, MA), passed through the MountainSort pipeline, and the sorted spikes were saved as text files for further analysis. Spike sorting using the manual method was completed using Spike2 (version 8, Cambridge Electronic Design, Cambridge, UK) on a PC running Windows 10 (Microsoft, Redmond, WA). Sorted spikes were analyzed using NeuroExplorer version 5 (Plexon, Dallas, TX) and MATLAB on a PC running Windows 10. Final results were processed in MATLAB, Excel 2013 (Microsoft), and Origin 2019 (OriginLab, Northampton, MA).

### Statistical analysis

Data are presented as mean ± standard deviation (SD), or median and quartiles (Q1 and Q3), as indicated. To examine statistical significance, which was defined as *P* < 0.05, we used two-sample ANOVA and Student’s t-test in samples that were normally distributed. Normality was assessed using the Shapiro-Wilk normality test. We also used nonparametric tests, namely Mann-Whitney U test and Kolmogorov-Smirnov test, in samples that were not normally distributed. All statistical tests were performed in OriginPro software (version 2021b; OriginLab Corporation, Northampton, MA).

## Results

The analysis process for neural data consists of multiple steps, using different software packages as required. A central goal of this work is to provide a roadmap for analysis of single-unit data, with a focus on how we have concatenated the Cheetah system with MATLAB and the automated spike sorting technology, MountainSort. The overall process begins with the acquisition of neural data, followed by spike sorting, and finally, the computation of spike parameters which is done in a manner specific to the phenomenon being studied. The code used in this study can be found at: GitHub: spike_sorting n.d..

### Description of the cheetah file formats

**Fig. 1:**
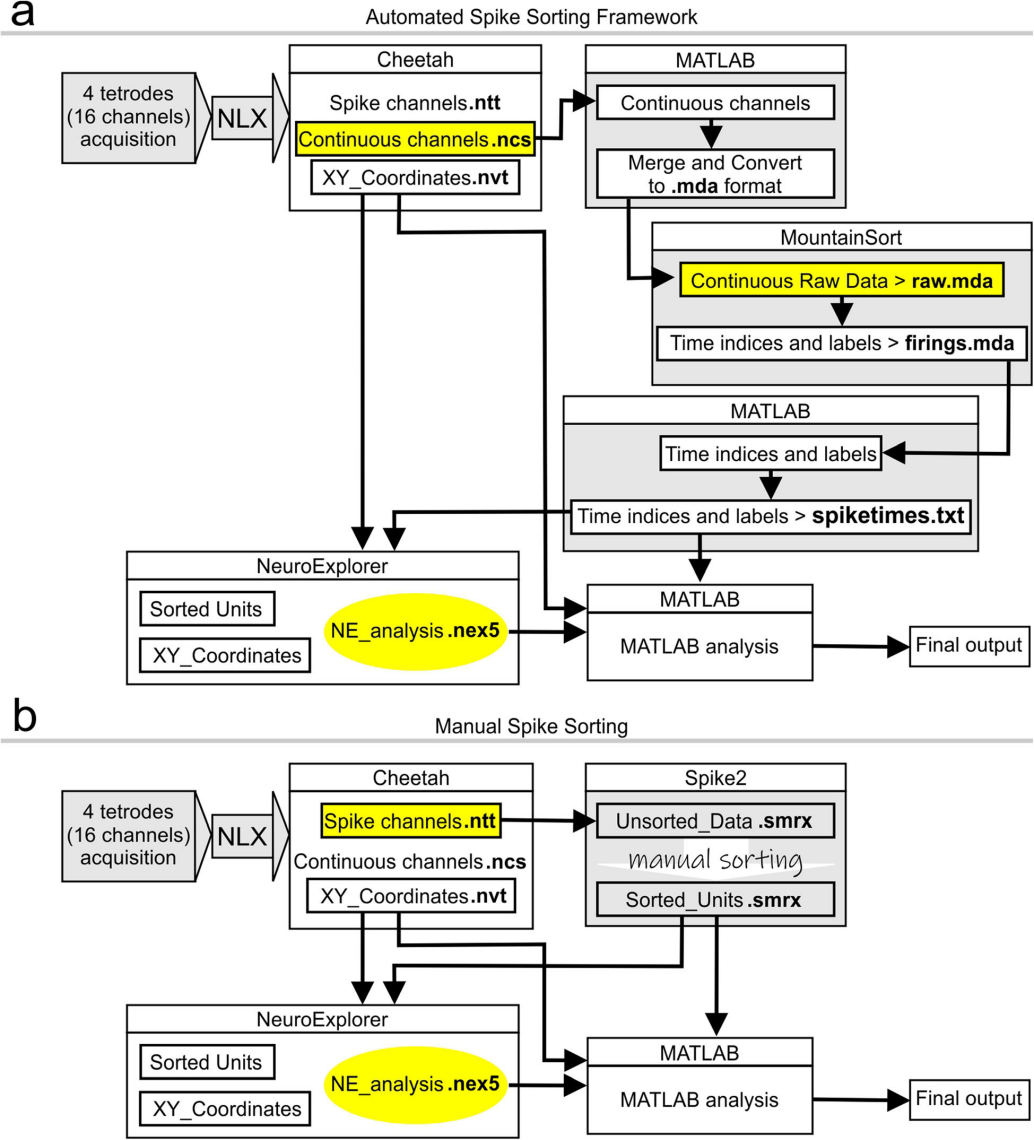
Overview of data analysis procedure. **a,** Automated spike sorting using MountainSort. Continuously sampled signals are collected in Cheetah, prepared for the MountainSort pipeline in MATLAB, sorted in MountainSort, and finally passed through MATLAB again prior to final analysis. **b,** Manual spike sorting using Spike2. Discrete spike channels are imported into Spike2, where all preprocessing takes place. Exported spikes are used for final analysis

The Cheetah system generates three groups of data (Fig. 1). The continuous datastream is saved in the files named CSC1.ncs up to CSC16.ncs (Neuralynx continuously sampled), which comprise the voltage at every timestamp (sample rate, 30 kHz) for each separate channel. The spike datastream is saved in the files named TT1.ntt up to TT4.ntt (Neuralynx tetrode), in which each file represents a tetrode and contains the spike information across the four channels of a particular tetrode. The XY datastream is saved in the file VT1.nvt (Neuralynx video tracking), which contains the position of an LED mounted onto the headstage on the animal’s head.

### Overview of the spike sorting methods

Both the automated spike sorting framework and manual spike sorting method follow similar overall trajectories. The raw data are saved by the Cheetah system, then brought into the spike sorting package, in which the spikes are sorted and exported for final analysis in NeuroExplorer and MATLAB, depending on the parameter in question (Fig. 1).

For the automated spike sorting framework, only the continuous channels are used (Fig. 1a). The ncs files are imported into MATLAB, they are merged, and then converted to the mda format (e.g., raw.mda). The files are then passed through MountainSort where pre-processing and spike sorting occur. Finally, the sorted spikes are saved as text files (e.g., spiketimes.txt) which allows for easy importing into most software packages. We use NeuroExplorer and MATLAB to analyze sorted data. Within NeuroExplorer, the analysis of spikes, along with the position data from the video feed (e.g., VT1.nvt), can be used to generate place field maps, autocorrelograms, and many other visualizations. From that point, the data can be exported into MATLAB for quantitative analysis such as calculation of place field areas or spatial information. For other forms of analysis, sorted spikes and position data are imported directly into MATLAB without passing through NeuroExplorer. Note that in order to import spikes from spiketimes.txt into NeuroExplorer, the correct option for importing data must be set. This can be done as follows: Open NeuroExplorer. Under the View tab, click on Data Import Options. In the window, click on the File Extensions box. Scroll down to txt and select the following option: Text File (pairs<channel> < timestamp>), and then click OK to exit the window.

For the manual spike sorting method (Fig. 1b), only the discrete spike channels from Cheetah are imported straight into Spike2 where file merging, spike sorting, and exporting of sorted units are completed. Our team has historically used manual spike sorting platforms such as Offline Sorter or KlustaKwik (Chang and Huerta, 2012; Faust, et al., 2013). In recent years, we have exclusively used Spike2 which has proven to be a versatile software for sorting and visualizing spike data. In Spike2, we use PCA to generate the components for cluster cutting. The spikes can be easily viewed as either overlaid waveforms or as points across time. If different recording sessions from the same day need to be merged together for sorting and then split back into the original sessions, this is all done within the Spike2 environment. Following completion of manual sorting, the spikes are exported for downstream analysis.

### Data preparation for MountainSort

**Fig. 2:**
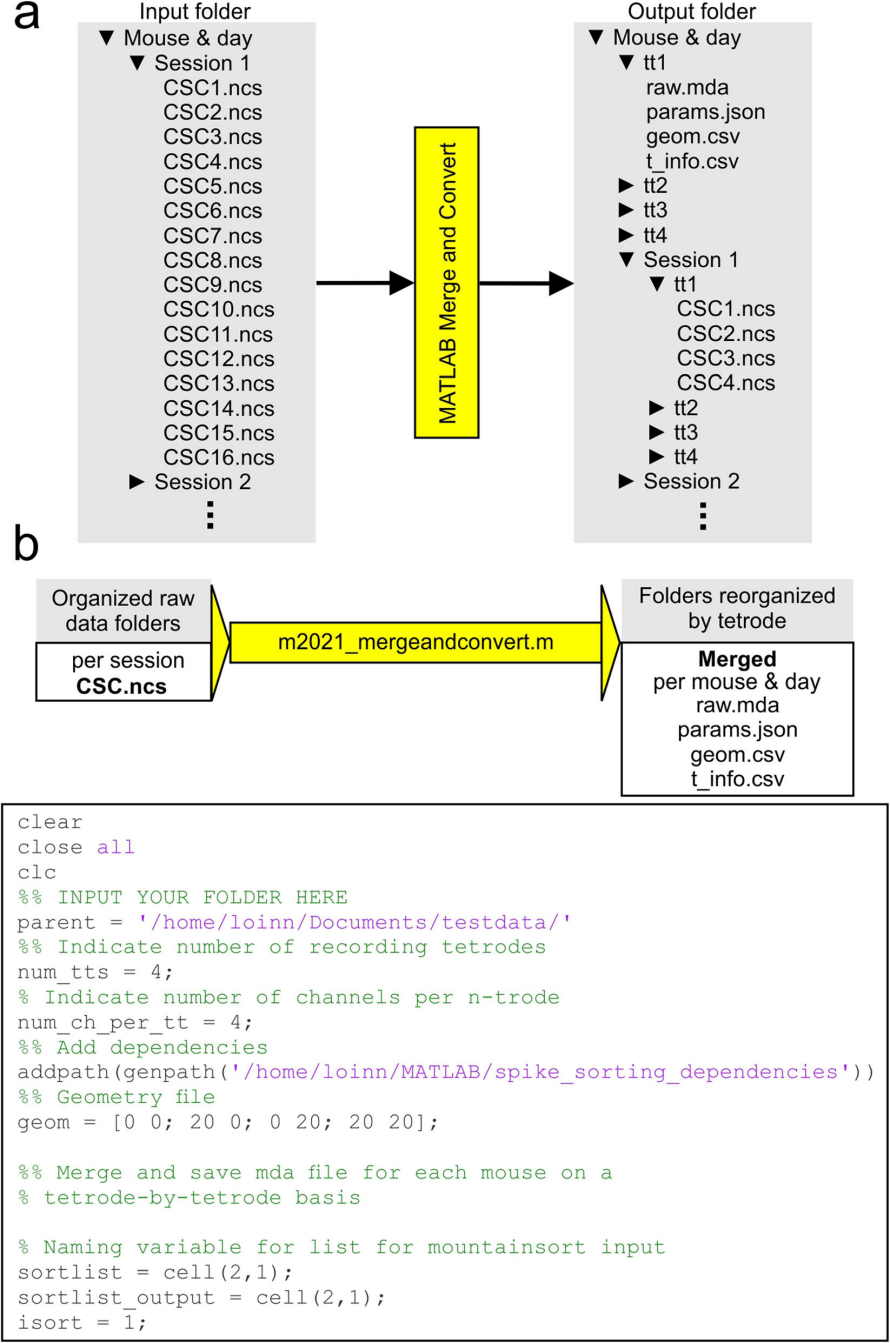
Merging and converting data prior to automated spike sorting. **a,** Organization and preparation of data for MountainSort pipeline. Sessions from the same mouse on the same day are placed into the same folder, with the data files organized in each session folder just as they are saved by Cheetah. The parent folder containing all of the ‘mouse & day’ subfolders is input into MATLAB to merge and convert the data. The output files are organized by tetrode, and saved in the mouse & day folder as they contain the signals acquired for all sessions within the folder. **b,** The m2021_mergeandconvert.m script imports the CSC.ncs files and saves the merged data as raw.mda. Three other files are saved; params.json contains the sample rate and spike direction, geom.csv indicates the tetrode geometry, and t_info.csv contains the start and end times of each recording that are used to split the merged files for session-by-session analysis. The lower panel depicts the m2021_mergeandconvert.m script to be run

The automated spike sorting process begins with the 16 continuously-sampled channels which are saved in the ncs format (Fig. 2a). In principle, these files contain the information about local field potential and the spike signals, but the spikes have not been isolated from the rest of the recording yet. The continuous channels are imported into MATLAB and the data files are merged according to the principle that all the recording sessions from a given mouse, obtained in the same day, are joined together. The merged files are then converted from the Neuralynx ncs data format into the mda (multi-dimensional array) format, which is compatible with MountainSort. Merging files is done so that spikes which may originate from the same neurons, can be clustered together, with the use of the m2021_mergeandconvert.m script (Fig. 2). This script was designed with tetrodes in mind, and the number of tetrodes used in the recording can be specified at the top of the script where indicated. If other recording configurations are used, the number of channels per n-trode may be altered as well, with 4 channels being equivalent to a tetrode. The input for this script follows a simple organization scheme. All of the experiments to be sorted need be placed into a single parent folder. Within the parent folder, recording sessions from the same mouse on the same day are grouped into individual ‘mouse & day’ folders (Fig. 2). Recording files go in the session folder with no additional subfolders, as is saved by default by the Cheetah system. Next, the m2021_mergeandconvert.m script is opened in MATLAB. The line asking for the parent folder needs to be changed to reflect the location of the parent folder stated above. Both the m_2021_mergeandconvert.m and m2021_ms_out_timestamps.m scripts require the folder spike_sorting_dependencies to be included in the MATLAB file path.

The m2021_mergeandconvert.m script first reorganizes the continuous data on a tetrode-by-tetrode basis so that each of the 4 recording channels from each tetrode is placed into an individual subfolder. Next, the ncs files (CSC1.ncs and so on) are imported into the MATLAB environment as variables using the mex files provided by Neuralynx. For each mouse & day folder, the raw continuous channels are imported and merged into a single [1 X M] variable. These files are then saved into the MountainSort-compatible format, as raw.mda, in which each file contains the 4 channels of a single tetrode for an entire mouse & day unit of recording. The raw.mda files are placed into folders created for each tetrode (e.g., tt1, tt2, tt3, tt4) in the mouse & day folder (Fig. 2a). Along with the mda files, three other files are generated for each tetrode. The first file, geom.csv, contains the electrode geometry for the recording, which is set to resemble the tetrode recording configuration. The second file, params.json, contains the sampling rate of the recording (in our case, 30 kHz) as well as the direction of spike occurrences (positive or negative). Both files are necessary for MountainSort. The third file, t_info.csv, gives the start and end time of each recording session for the mouse & day, and it is used (after MountainSort) to separate the merged files back into their original recording sessions (Fig. 2b). For each run of the m2021_mergeandconvert script, a file titled runsort.sh is generated. This file contains the file paths needed to input each tetrode into MountainSort, as well as paths for the output files generated by MountainSort.

**Fig. 3:**
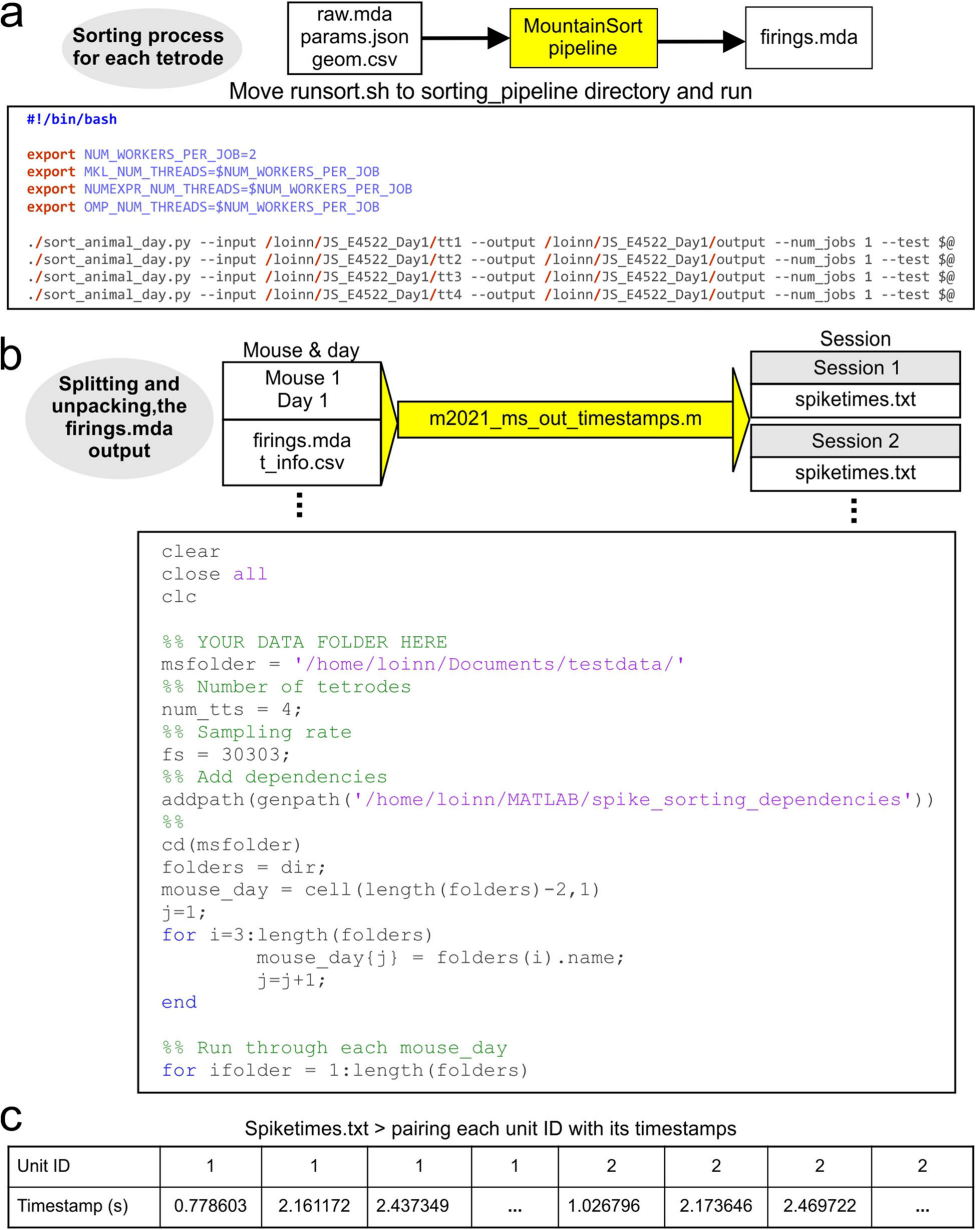
Automated sorting framework. **a,** The files raw.mda, params.json, and geom.csv are imported into the MountainSort pipeline, which includes both pre-processing and spike sorting. The MountainSort pipeline outputs firings.mda files, which contain the unit IDs and timestamps of sorted spikes. The designated input and output folders are entered into the runsort.sh script, as depicted in the bottom of the panel. **b,** Using the m2021_ms_out_timestamps.m script, sorted files are split back up into the original recording sessions according to the t_info.csv file. Split files are saved as spiketimes.txt, for easy transfer to other software package for final analysis. The m2021_ms_out_timestamps.m script is shown in the bottom of the panel. **c,** Output spiketimes.txt files are formatted as two columns of unit ID, timestamp pairs

After running the m2021_mergeandconvert script, the data are ready for the spike sorting pipeline. Prior to sorting any experiments, MountainSort needs to be installed according to: GitHub: sorting_pipeline n.d.. After installation, this pipeline can be executed by moving the runsort.sh file into the sorting_pipeline folder, and executing this file in the terminal. The runsort.sh file then leads MountainSort to input the raw.mda, params.json, and geom.csv files, run the pipeline, and generate a firings.mda output for each tetrode, which contains the timestamps for the firing times for each sorted unit (Fig. 3a).

### Interpretation of the sorted spike data

Following MountainSort, the firings.mda files are saved in an appropriate format for further analysis. This is achieved by running the m2021_ms_out_timestamps.m script. The merged and sorted files are first split up into sessions as originally recorded (Fig. 3b). To ensure compatibility with multiple analysis programs, the output is saved as a text file titled spiketimes.txt. This file contains two columns, which make up [unit ID, timestamp] pairs (Fig. 3c). This file structure can be imported into a variety of analysis packages including MATLAB and NeuroExplorer. The waveforms are not exported with this dataset, allowing for fast computation and small file sizes. If waveforms need to be viewed, a MATLAB-based waveform viewer is available at: GitHub: matlab_waveforms n.d..

The waveforms are found by matching the timestamp to the raw.mda data for each isolated unit. From there, the waveforms are saved as mat files in MATLAB and can be plotted as overlaid individual waveforms, or as the average waveform for each single-unit. Other waveform viewing options are currently being developed and may be available on the GitHub page for the Flatiron Institute: GitHub: Flatiron Institute n.d..

### Validation of the automated framework with CA1 recordings

**Fig. 4:**
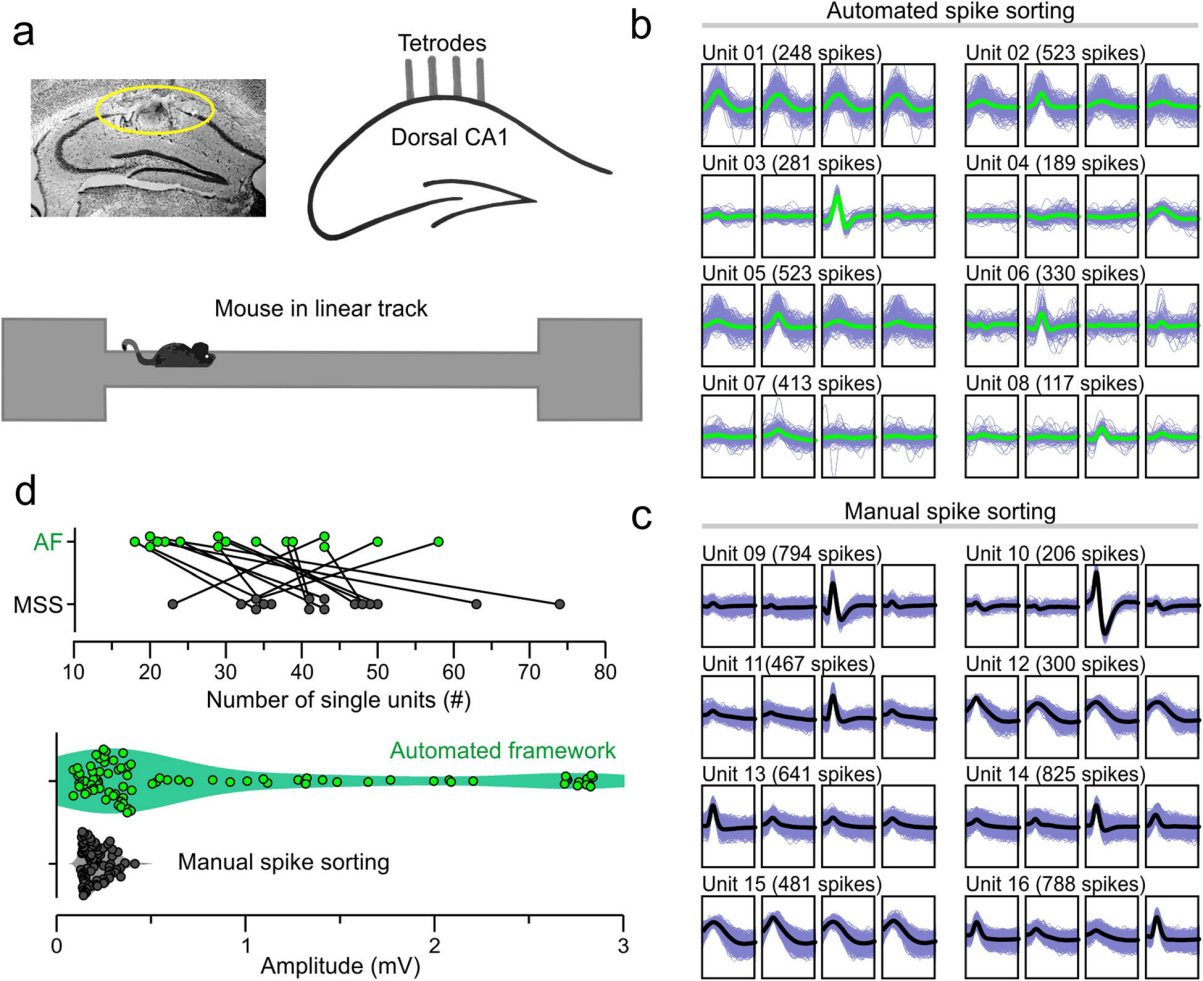
Validation of the automated framework with hippocampal recordings. **a,** Schematic depicting tetrode placement in the CA1 region (indicated by yellow oval) and recording paradigm in the linear track. **b,** Representative waveform overlays of single-units sorted using the automated framework. **c,** Waveform overlays of single-units sorted using the manual method. **d,** ***Top,*** number of single-units sorted per recording session using the automated framework (AF, green) or manual spike sorting (MSS, black). ***Bottom,*** amplitudes of mean waveforms sorted using the automated framework (green) or manual method (black)

Neural signals were recorded in the *stratum pyramidale* of CA1 in mice (*n* = 4) that were running along a linear track (Fig. 4a). The spiking activity was used to compare the output of the automated framework against a manual sorting method. We show several examples of single-units, chosen randomly, which were obtained with the MountainSort pipeline (Fig. 4b) and manually-sorted with Spike2 (Fig. 4c). Manual spike sorting was performed by experienced operators and the final results were checked for quality by two independent observers. Comparison of single-units sorted automatically and manually, during 16 recording sessions (Fig. 4d), revealed that the total number of single-units per session was not significantly different between groups (Fig. 4d**, top**; automated = 32.31 ± 11.85 [mean ± SD], range = 18–58; manual = 43.31 ± 12.35, range = 23–74; *t* = 2.13, *P* = 0.05, paired *t* test). However, comparison of the spike amplitudes, defined as the peak-to-peak voltage of the averaged waveform from each sorted unit, showed that the automated framework sorted single-units of significantly higher amplitude than the manual method (Fig. 4d**, bottom**; automated: median = 0.35, Q1–Q3 = 0.22–1.32; manual: median = 0.19, Q1–Q3 = 0.15–0.25; *d* = 0.55, *Z* = 3.55, *P* = 3.49 × 10^− 12^, Kolmogorov-Smirnov test).

**Fig. 5:**
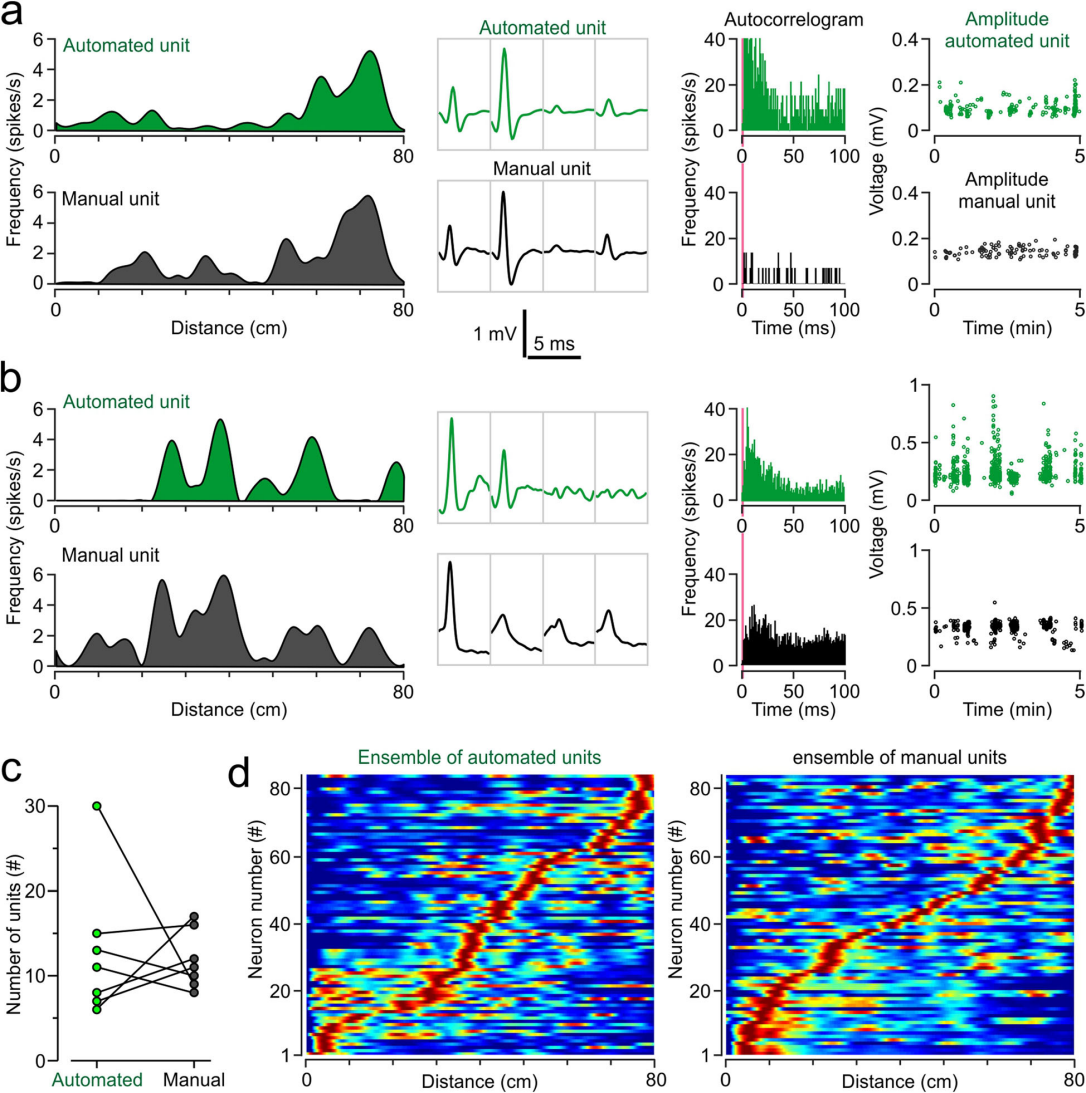
Hippocampal CA1 recordings in freely-moving mice. **a, b,** Representative single-units showing the firing rates along the length of the track (left panels), average waveform in each channel of a tetrode (next-to-left panels), autocorrelogram (next-to-right panels; pink lines represent refractory period), and amplitudes of spikes over time (right panels). Automated units are displayed in green and manual units are shown in black. **a,** Representative single-units likely representing the same putative neuron sorted with automated and manual methods. **b,** Similar units likely to represent different neurons across automated and manual methods. **c,** Number of single-units per recording session using the automated framework (green) or the manual method (black). **d,** Population of place cells recorded from mice (*n* = 4) running the linear track. Each row represents normalized spike activity of one single-unit, where rows are organized by the position of the peak firing rate for each unit along the linear track. Sorted populations of place cells appear similar across automated and manual methods

We used the place cell properties of the hippocampal single-units sorted automatically (MountainSort pipeline) and manually (using Spike2) for direct comparison of the output from both methods. For illustrative purposes, two pairs of single-units with place fields in close proximity across methods were selected (Fig. 5a, b). In one case (Fig. 5a), the two place cells have similar firing rates as a function of position of the mouse along the linear track (Fig. 5a**, left**) and display a well-defined place field (at ~ 70-cm on the track), with the peak firing rate occurring as the mouse moves from right to left. Moreover, their waveforms (Fig. 5a**, next-to-left**) are highly similar across the 4 channels of the tetrode when comparing the automated and the manual traces. Their autocorrelograms (Fig. 5a**, next-to-right**) show few refractory period violations but appear different depending on the sorting method, which is likely the result of MountainSort clustering more spikes into the single-unit when compared to manual sorting operators. Analysis of all spike events during a 5-min period shows that the spike amplitudes for the two sets are statistically different (Fig. 5a**, right;** automated = 0.103 ± 0.03 [mean ± SD]; manual = 0.164 ± 0.018; *F* = 2.81, *P* = 6.65 × 10^− 9^, two-sample ANOVA). Notably, the spikes appear to fire in bursts, as would be expected of hippocampal place cells, with the automated unit showing more pronounced bursting, and also more variance, than the manual unit. This example suggests that the automated framework is more accepting toward clustering spikes of various amplitudes into a single-unit when compared to the manual method. The second example (Fig. 5b) depicts two place cells with multiple place fields, as revealed in their firing profiles (Fig. 5b**, left**). The primary place field is near the center of the track (20–40 cm), when the mouse moves from right to left, but relatively high firing rates occur in other regions of the track as well. Although the main place field is similar across sorting methods, the large differences in extraneous activity suggest that the single-units are not optimally clustered. The waveforms (Fig. 5b**, next-to-left**) show high-amplitude traces in channels 1 and 2, but the manually-sorted waveforms also have high amplitudes in channels 3 and 4 compared to the automated traces. The autocorrelograms (Fig. 5b**, next-to-right**) show few refractory period violations but the shapes are different, with the automated unit featuring more firing close to zero, which suggests a greater tendency to burst, whereas the manual unit displays a firing pattern that is spread through the autocorrelogram. Analysis of all spike events during a 5-min period of recording shows that the two sets are statistically different (Fig. 5b**, right**; automated = 0.231 ± 0.092 [mean ± SD]; manual = 0.329 ± 0.048; *F* = 3.575, *P* = 1.65 × 10^− 46^, two-sample ANOVA). The bursting nature of the single-units is evident in both methods, but there is more variance in the amplitude of the automated unit. It is possible that in both cases, spikes arising from different neurons were clustered as the same unit. The higher extraneous firing in the manual unit, as well as the differences in the waveforms across sorting methods, strongly suggest that more inappropriate spikes were included in the manual method compared with automated sorting.

When counting the number of single-units classified as place cells across 7 sessions in the linear track, we found that the automated and manual sorting methods yielded similar quantities of single-units per recording (Fig. 5c**;** automated: median = 11, Q1–Q3 = 7–15; manual: median = 11, Q1–Q3 = 9–16; *U* = 21, *Z* = 0.38, *P* = 0.7, Mann-Whitney U test). Only 2 sessions behaved as outliers, with the number of single-units varying substantially across methods (Fig. 5c**;** automated with 30 vs. manual with 9; automated with 6 vs. manual with 17). While there are similarities and differences among spikes sorted with the two methods, neurons act together in groups, so it is important to view results at the ensemble level as well as that of the individual neuron. By normalizing the firing rates of each unit and sorting all units by the location of their peak firing rate, the activity of place cells in the linear track can be viewed as an ensemble of units (Fig. 5d). We implanted 4 mice and recorded as they ran the length of the track and generated place cell ensembles using each sorting method. Here we find that, at the ensemble level, the activity of the place cells appears similar across both methods. Remarkably, in the ‘automated spike sorting’ ensemble, place fields appear better isolated compared to the ‘manual spike sorting’ ensemble (Fig. 5d), as suggested by the reduced extraneous activity outside of the main place fields for each unit. Importantly, both sorting paradigms result in ensembles covering the full length of the track.

### Validation of the automated framework with PL cortex recordings

**Fig. 6:**
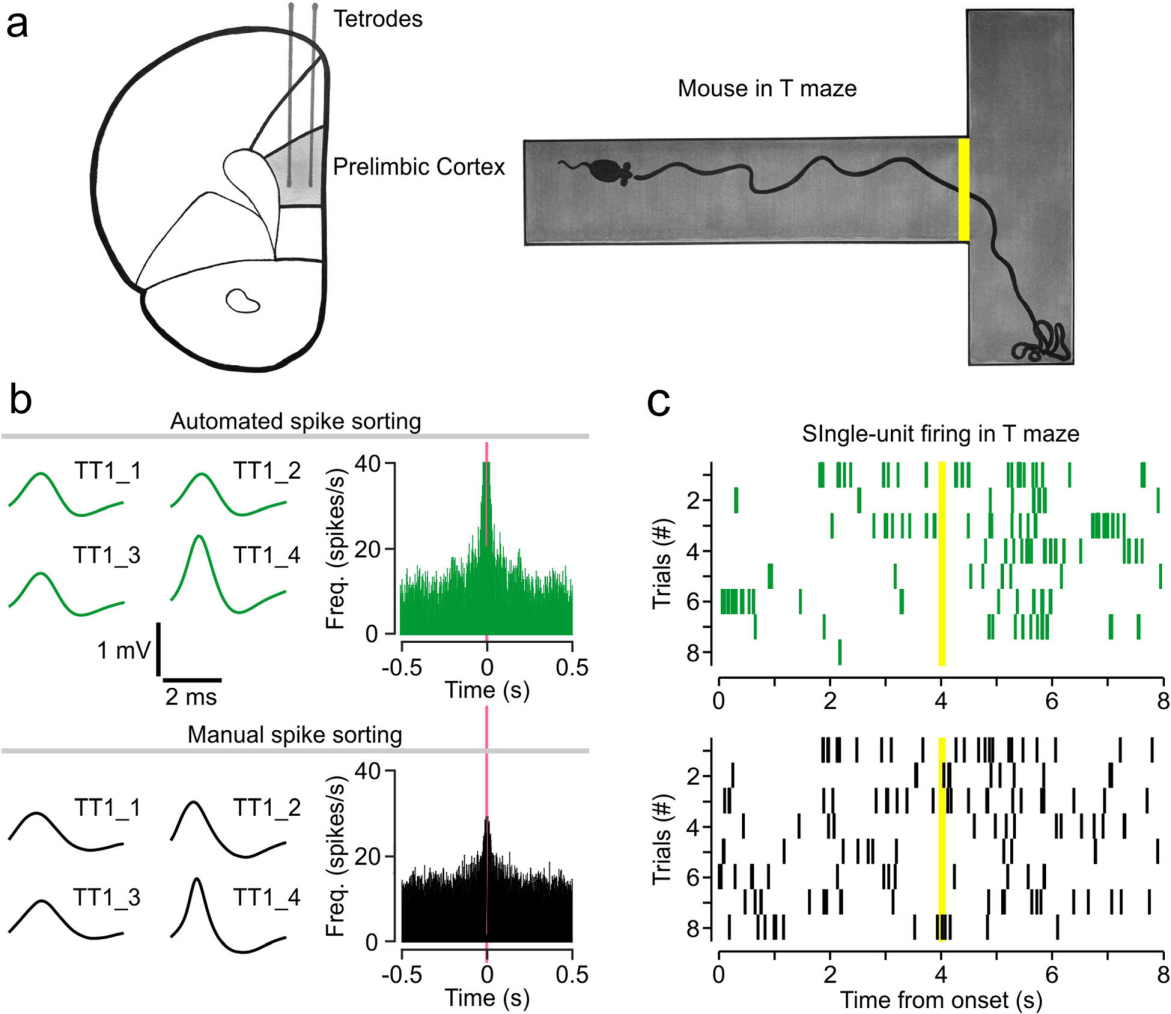
Prelimbic cortex recordings in freely-moving mice. **a,** Schematic depicting tetrode placement in the PL cortex and recording paradigm in the T maze. The yellow line indicates the point at which the mouse chooses to go left or right. **b,** Representative average waveforms and autocorrelograms of sorted units, likely representing the same putative neuron, across automated and manual methods; TT1_1, and so on, indicate tetrode channel numbers. The pink line represents the refractory period. **c,** Raster plots for single-units in panel b. Each row represents a trial in the T maze and each tick is a spike. Notice that the trials are aligned to the timepoint indicated by the yellow line (4 s), which corresponds to the moment that the mouse arrives at the intersection of the stem with the arms of the T maze in each trial (as in panel a)

We sought to compare the automated framework (MountainSort pipeline) and a manual sorting method (Spike2) using recordings from the PL cortex, a region located within the medial prefrontal cortex. Prior to surgery, the mouse was trained to go to the right arm in the T maze. Tetrodes were implanted into the PL cortex, and the freely-moving mouse was tested in the T maze (Fig. 6a). We examined sorted units based on their activity as the animal navigated from the start point (in the stem) toward the reward (end of the arm). For illustrative purposes, a pair of single-units that seem to have similar properties across methods are shown (Fig. 6b). In this example, the automated unit refers to a neuron that is sorted using MountainSort (Fig. 6b**, top**) and the manual unit is sorted with the manual method (Fig. 6b**, bottom**). The waveforms from the automated unit are nearly identical to the waveforms of the manual unit, and the autocorrelograms are similar as well, although the automated unit displays more firing and bursting (Fig. 6b). By plotting the spike activity of the example units as trial-by-trial raster plots, one can observe their dynamic activity (Fig. 6c). Each row of the raster represents the firing of the unit during a trial in the T maze, and the center of the plot corresponds to the time when the mouse is at the intersection of the stem and the arms of the T maze (yellow line in Fig. 6a, c). Statistical comparison shows that the numbers of isolated spikes per trial are not significantly different between the two sets (Fig. 6c**;** automated: median = 27.5, Q1–Q3 = 14.25–42.5; manual: median = 15.5, Q1–Q3 = 13.25–23.5; *U* = 45, *Z* = 1.315, *P* = 0.189, Mann-Whitney U test). Notably, the automated unit shows quite a similar pattern of activity across multiple trials, which highlights the reliability of the automated method in isolating a well-behaved unit. In contrast, the manual unit displays a more widespread pattern of spiking across the trials, which would be indicative of a poorly-isolated neuron.

## Discussion

We have designed a novel framework to smoothly combine the acquisition of neuronal signals from tetrode recordings of freely-moving mice, using the Cheetah system (Neuralynx), to the automated spike sorting pipeline MountainSort (Chung et al., 2017). It is clear that automated spike sorting has become a necessity for medium and large-scale extracellular neural recordings, as it involves the extraction of (ideally) all the action potentials generated by an individual neuron from an ocean of activity in the extracellular recordings. We provide a detailed roadmap of the steps, from data acquisition to file managing between the different software platforms (Cheetah, MATLAB, MountainSort, and NeuroExplorer). In short, our solution provides full integration of MountainSort-based spike sorting to Cheetah-based acquisition of neuronal signals in mice. The framework presented in this study is not intended to be generalizable across multiple acquisition systems and spike sorters, but rather it is designed to be a specific solution for users of Neuralynx systems. The advantage of our simple approach is that in a single run, multiple recordings can be merged, sorted, and exported, with minimal user input or troubleshooting. Moreover, the framework does not require the end-user to create any new scripts of their own, which is a common necessity with other spike sorting frameworks.

The framework is intended to be completely compatible with existing analysis software; therefore, we convert sorted spikes from the mda format (used by MountainSort) to a simple txt format. While there is indeed a network of analysis tools being developed around MountainSort, these packages are still in the development phase. Furthermore, many established labs need a way to seamlessly implement state-of-the-art automated spike sorting methods in a manner compatible with existing analysis processes. In our case, we use NeuroExplorer and MATLAB, both of which are widely used programs for the analysis of neural data. Thus, we have created an original end-to-end framework to integrate the automated spike sorting technology of MountainSort with Cheetah-acquired recordings into a data analysis framework consisting of widely-used software packages.

There are several benefits to using the automated pipeline. Critically, automated spike sorting provides a repeatable and objective methodology, in contrast to manual methods which might be quite subjective. Manually sorting spikes yields variable datasets among different operators, even when the operators have significant experience with the technique. Another important benefit regards to the amount of time taken to sort datasets of almost any size. Manually sorting is a slow and user-intensive process. Each recording can take a period of user-input time ranging from several minutes up to a few hours to finish, making for a time-intensive process. In contrast, using the automated framework, multiple recordings can be quickly sorted in a single run. In this scenario, MountainSort processes each recording in a period of time shorter than the recording itself, but the user does not need to be actively working during this time period. The user only needs to set up the files to be recorded, reducing the user-input time to mere minutes per recording.

We have compared the results of the automated spike sorting framework with a manual spike sorting platform we have used for several years in our laboratory. Using either approach, we can isolate single-unit activity in two different brain regions, the hippocampus and the PL cortex. When we compared the number of units sorted per session, we found similar numbers of automated units and manual units for most sessions, but there were some exceptions. In particular, there were two sessions that stood out as outliers. For one of these outliers, the automated framework sorted more single-units, whereas for the other, the manual method sorted more single-units. While there may be differences at the level of each single-unit, we can identify units isolated with similar waveforms across both algorithms. These units also show similar activity in behaviorally relevant tasks such as the linear track or T maze. Furthermore, when viewed at the ensemble level, the datasets look largely similar with only slight variations in the precision of firing.

We think that automated spike sorting platforms are poised to become a critical component in the neuroscientist’s toolkit, considering that within the next decade, we will very likely experience an exponential increase in the size of the recording arrays (Alivisatos et al. 2013; Steinmetz et al., 2021). With the next generation of brain probes including up to thousands of recording sites, the sheer complexity of the datasets is bound to be too large for manual sorting, or even semi-automated methods, to be capable of processing them. It seems apparent that automated spike sorting needs to become easy to use and properly validated across different acquisition setups, brain regions and mammalian species. In this respect, we have presented a complete automated framework for sorting neural spike data acquired from Neuralynx-based systems. Our framework is easy to implement and provides a high-throughput solution. We predict that within the broad field of bioelectronic medicine, those teams that incorporate high-density neural recording devices to their armamentarium might find our framework quite valuable as they expand their analytical footprint.

## Conclusions

- We present a complete framework for automated spike sorting using the MountainSort package, with all the code freely-available in the GitHub repository.
- The toolset delivers automatically-sorted spikes from recordings obtained with a Cheetah-based system, and exports the sorted spikes in a format which is highly compatible with existing data analysis routines.
- Integration of automated spike sorting software is a critical step in the analysis of neural data, and becomes increasingly important as the size and complexity of datasets continues to increase with new electrode technology.
- An improved understanding of the neural signals obtained with tetrode recordings within the brain will be paramount for any bioelectronic approaches targeting brain systems.

## Data Availability

The datasets used and analyzed during the current study are available from the corresponding author on reasonable request. Code used in this manuscript may be found at the following addresses: **https://github.com/HuertaLab/spike_sorting****.** DOI: 10.5281/zenodo.5537196 **https://github.com/HuertaLab/spike_sorting/tree/main/sorting_pipeline** **https://github.com/HuertaLab/spike_sorting/tree/main/matlab_waveforms**

## Abbreviations

AP: anterior/posterior
CA1: *Cornus Ammonis 1*
cm: centimeter
Hz: hertz
kg: kilogram
kHz: kilohertz
kΩ: kilo-ohms
LED: light-emitting diode
mg: milligram
ML: medial/lateral
min: minute
mV: millivolt
PL: prelimbic cortex
s: second
